# The AI-aging-enterprise: a political economy of aging and artificial intelligence

**DOI:** 10.1093/geront/gnaf262

**Published:** 2025-11-21

**Authors:** Vera Gallistl, Clara Berridge, Muneeb Ul Lateef Banday, Justyna Stypinska, Anita Ho, Robin N Brewer, Alisa Grigorovich, Alexander Peine, Anna Wanka

**Affiliations:** Center for Gerontology and Health Research, Department General Health Studies, Karl Landsteiner University of Health Sciences, Krems, Austria; School of Social Work, University of Washington, Seattle, Washington, United States; Interdisciplinary Center for Gender Studies (IZFG), University of Bern, Bern, Switzerland; Organisational Behaviour and Human Resource Management, Goa Institute of Management, Goa, India; Berlin Social Science Center (WZB), Berlin, Germany; Bioethics Program, University of California, San Francisco, California, United States; School of Information, University of Michigan, Ann Arbor, Michigan, United States; Recreation and Leisure Studies, Brock University, St Catharines, Ontario, Canada; Faculty of Humanities, Open University of the Netherlands, Heerlen, The Netherlands; Department for Educational Sciences, Goethe University Frankfurt, Frankfurt/Main, Hessen, Germany

**Keywords:** Critical gerontology, Material gerontology, Ethics, Interventionism, Technology

## Abstract

The current discourse on artificial intelligence (AI) in gerontology remains mostly on an interventionist level and focused on solving problems faced by individuals, leaving the wider social conditions that shape the relationship between aging and AI out of view. The considerable accumulation of power, particularly for technology development companies, in the development and implementation of AI, however, calls for a deeper and more complex analysis of the relationships between AI, older adults and the “aging enterprise.” Building on the classical political economy of aging, and expanding it with concepts from material gerontology, we propose a “political economy of aging and AI” as a conceptual tool to analyze the relationships between (older) individuals, social and political structures, and (technological) materialities. We exemplify how such a political economy perspective enables a more complex consideration of the current discourse on (a) AI solutions, (b) AI ethics, and (c) AI policies. We close by summarizing our understanding of a political economy of aging and AI that centers materialities as the critical-gerontological focus of analysis and by outlining calls for action toward strengthening the power of older adults in the emerging AI-aging-enterprise.

Political economy perspectives on aging have been a vital part of critical gerontological debates in the past—from pensions to civic engagement (Markson & Williamson, 1984; Martinson & Minkler, 2006)—but have hardly yet been used to theorize the current development of artificial intelligence (AI) as gerontechnologies. Contemporary gerontological debates on AI are primarily concerned with the use of AI by older individuals and ethical challenges that accompany AI implementation (Gallistl et al., 2024). With that, the gerontological discourse follows an interventionist logic that has been well established in gerontological research on technology (Peine & Neven, 2019). Problems posed by different types of social and political structures have received limited attention in the field’s literature, particularly problems connected to social inequalities and the uneven distribution of power in datafied, aging societies, and economies (Nayak & Walton, 2024).

Following Suchman (2023), AI is not a thing in and of itself, but rather, can be thought of as “a label for currently dominant computational techniques and technologies that extract statistical correlations (designated as patterns) from large datasets, based on the adjustment of relevant parameters” (para 7). The fast-paced development of these technologies and techniques that are referred to as AI represents not only technological advancements, but also economic opportunities and have been accompanied by a considerate accumulation of wealth and power, especially for technology development companies. AI development is projected to continue to affect governance, economies, and democracies across the globe (Nayak & Walton, 2024), particularly through creation of new forms of capital and dynamics of wealth accumulation. These structural issues that shape the relationship between older adults and AI systems require going beyond an interventionist approach toward a structural analysis of the complex relationships of power inherent in the relationships between older adults and AI. It also requires critical gerontological theory to think deeper about the role of (technological) materialities in governing, suppressing, and marginalizing old age.

The aim of this article is to put forward such a structural way of thinking about the relationship between aging and AI. Building on classical concepts from critical gerontology, and expanding it with new advances in material gerontology, we propose a “political economy of aging and AI” as a conceptual tool to rethink AI-aging-relationships beyond an interventionist logic.

In the following, we lay out basic principles of a political economy (PE) of aging approach and then ask how they can be applied to critically think through the current gerontological discourse on (a) AI solutions, (b) AI ethics, and (c) AI policies. In the concluding discussion, we put forward main concepts that we think can fruitfully expand a political economy perspective on the current AI hype and movement. Aiming to apply as well as expand classical political economy perspectives, we propose major concepts and fields of study for a critical gerontological engagement with AI going forward.

## The political economy of aging

The PE approach in gerontology was developed in the 1980s. Its defining distinction as a gerontological theory is how it positions the aging not as an individual, but as a structural phenomenon. It constitutes a critical perspective on the “broad social, economic, and political factors and structural arrangements that are integral to and that produce old age” (Estes, 2001). Scholars like Carroll Estes, Peter Townsend, Alan Walker, and Chris Phillipson, criticized prevailing gerontological theories for an overemphasis on the individual “as the primary unit of analysis” (Estes, 2001), and instead emphasized the sociohistorical, political, and economic structures in which people grow older, as well as ideas around what “older adults” are. Sparking an ongoing debate in the field, they criticized how individualist perspectives that center the aging individual contribute to unfairly placing responsibility on those individuals for their dependencies and vulnerabilities (Phillipson, 1991). Instead, PE approaches refocus on the structural conditions that produce those dependencies and vulnerabilities and the idea of aging as an inherently vulnerable stage of life.

Applied to technologies, a PE approach would offer critiques of the individualist perspectives from which technology and aging have largely been studied (Peine & Neven, 2019; Gallistl et al., 2024). Many studies in gerontology aim to understand individual acceptance, use, or nonuse of technologies by older adults (cf. Lipp & Peine, 2024), and focus on the individual, for example, by involving older adults in participatory technology design, at times with too little regard for the well-documented risks of overlooking power imbalances in technology co-design (Merkel & Kucharski, 2019). From a PE perspective, framing older adults primarily as technology users and targets of individual interventions directs the field’s attention away from a structural analysis of how aging and technology come together, which economic opportunities are created through these entanglements, who profits from these opportunities, and the power imbalances that exist between older adults and technology development companies.

Instead of such an individualistic perspective, PE approaches have traditionally understood technology both as a driving force behind capitalist modes of production and a potential to liberate workers from the drudgery of capitalist labor (Rammert, 2000; Rosenberg, 1982).^1^ This has led to an understanding of technological infrastructures as unspecific “material conditions” (Beetz, 2016) that humans find themselves in, are governed through and oppressed by—but analyzing, let al.ne governing technological change itself has remained outside the scope of critical-gerontological analysis for a long time. Such “old materialisms” (Hahmann & Höppner, 2025) largely argue in line with a technological determinism often accredited to Marx (see Wyatt, 2008): Older individuals are victims, so to speak, to the technological developments that determine the material conditions of growing older, while these developments themselves are taken to proceed on a logic of their own not related or made by human actions and interactions.^2^ This (technological) determinism often connected with PE approaches has also sparked critique, as authors note that PE approaches have overemphasized structures and sidelined the agency of (older) individuals to change these structures (for a critical discussion on the use of agency as a concept in gerontology, see Grenier & Phillipson, 2013).

In recent years, such old materialisms have been fruitfully expanded by a *new materialist stance* (Höppner, 2023), which underlines the “madeness” of materialities and is closer to Marx’s own take on technological change as collective, social processes and their interplay with economic and institutional environments. This stance highlights the human capacities that go into producing AI systems and the “material politics” (Kim, 2020) that are inscribed into AI in this production process. This *new materialist gerontology* offers a fruitful opportunity for a critical analysis of AI for two reasons: First, it echoes and integrates a larger epistemological turn in the social sciences and humanities, which granted materialities a more active and agentic role in the (un)making of social phenomena—including the making and unmaking of social inequalities. Criticizing existing social theories for their structural determinisms and an overall focus on human actors, human actions and human agency, new materialist theories bring a stronger focus to the material world—such as AI systems—in shaping social phenomena. Second, the material turn reacts to technological advancements to some extent, as it has been claimed that the turn toward more or less autonomous decision-making systems withhumanoid characteristics hasmade “a priori separations between humans and nonhumans difficult to sustain” (Hazard, 2019, 629). In line with these developments, new materialist gerontology has hence argued to grant nonhuman actors (such as technologies) a more active role in shaping the conditions that might victimize or marginalize older adults.

Instead of exploring how older adults are structurally determined by the technologies that surround them, a new materialist stance would hence critically explore how the phenomenon of aging itself is made through the entanglement of humans (such as older people) and materialities (such as AI systems). Hence, a new materialist stance toward AI might fruitfully expand a PE critique by asking, for example, how dependency, vulnerability and definitions of “old age” itself are made within those “aging assemblages” (Endter et al., 2024) that AI technologies are increasingly part of; how the boundary-making between dependent and independent, young and old, older (human) adults and new (nonhuman) technologies, are drawn; and who profits from drawing these distinctions.

In the following, we outline how a political economy perspective, informed by both new and old materialisms, might be applied to three pressing issues surrounding the relationship between aging and AI. We focus on gerontological discourses on (a) AI solutions, (b) AI ethics, and (c) AI policies. For each of these fields, we outline (a) how and why an individualistic perspective has been dominant, and (b) which additional insights and critiques a structural and materialist PE perspective might add.

## AI solutions

In 1979, Estes argued that the assumptions of decline produced aging as a “problem” of being “special, different and dependent” while at the same time making older people responsible for that problem. This problem framing made aging available for medicalization and economic investments and hence positioned growing older as a problem that could be solved through biomedical and technological interventions. This logic, Estes (1979) continued, produced the “aging enterprise,” a medical–industrial complex encompassing “programs, organizations, bureaucracies, interest groups, trade associations, providers, industries, and professionals” (Estes, 1979), all organized around commodifying aging solutions.

The rise of AI has intensified this discourse of gerontological solutionism, which posits technological interventions as optimum, efficient or the only solutions to the “problems” connected to population aging (Neven & Peine, 2017) that “once narrowly defined, are set to be narrowly solved” (Fletcher, 2025). This interventionism of AI in the context of aging (Neves et al., 2023), however, often disregards the social, material and political processes that position aging as a problem in the first place. It also tends to marginalize the voices of older adults in finding and implementing other—maybe not technologically supported—solutions to the challenges they experience in later life.

Further, AI solutionism tends to overlook the structural issues that accompany the implementation of AI. One of these structural issues concerns the (often not transparent) practices of datafication that go along with the deployment of AI, particularly in sensitive settings such as long-term care. Here, algorithmic technologies are often imagined as having potential to provide real-time information about patients and support care workers in deciding about relevant courses of action (Lukkien et al., 2024). What often remains undiscussed are processes of datafication that such systems require and how these format the knowledges that are regarded as legitimate for the governance and control of care and old age (Greubel, 2024). For example, Gallistl and von Laufenberg (2024) show how data used for AI development in long-term care is often synthetically created, rather than collected by engaging with the real-world experiences (and problems) of older adults in long term care. These practices ultimately position older adults as voiceless in the development and implementation of AI in care settings.

Berridge & Grigorovich (2022) observe that AI solutionism also appears to sideline the opportunity cost of investment in AI in care environments over attention to the structural causes of poor quality of care (e.g., understaffing and work overload). For example, Neves et al. (2023) show how AI in long-term care is often imagined as a set of promised solutions—particularly around monitoring and detection—that aim to increase the objectivity and effectiveness of care. How far AI systems are actually able to deliver on these promises of better quality and efficiency of care, however, is hardly questioned or evaluated. On the contrary, Gallistl and von Laufenberg (2023) argue that more care resources and staff capacity are needed to assist the implementation of AI in long-term care, ultimately leading to more costs for care home providers.

The implementation of AI thus risks further entrenching the marketization of long-term care (Corlet Walker et al., 2022) that profits from the collection of big data, uses it for algorithmically mediated or automated decision-making, and through this, ultimately prioritizes machine-readable knowledge as privileged modes of knowledge production about aging and care (Cozza, 2024). This marketization positions AI as an efficient solution for minimizing the costs of services for older populations (Fletcher, 2022). It also enables conditions for private businesses in the “medical–industrial complex” (Sharon, 2018) and makes older individuals responsible for investing in their care and enterprising management of their daily lives (Lipp & Peine, 2024).

A PE perspective, as we are putting forward in this article, asks what the modes and practices of profit generation are that underpin the development and implementation of AI as a solution for the diverse problems in the contexts of aging. For example, this includes making visible the situated materialities of AI systems—the data banks, the sensors, the processors, and hard drives—that represent to a large part the capital that is made available and increasing in scale in this regime of aging and AI. This also includes asking how data for the development and use for AI is collected, extracted and made into profit for companies in the form of data capital. Scholars have, for example, shown that the accumulation of this data capital often follows the principles of data extraction—wherein data are taken with little regard for consent and compensation (Sadowski, 2019)—and hence positions older adults as valuable vessels of data, rather than informed and empowered subjects in relation to AI (Gallistl & von Laufenberg, 2024).

## AI ethics

AI solutionism positions AI as something that will inevitably transform responses to the perceived problems of population aging. Current gerontology discourse often narrows the question of if older adults want to use AI to an ethical and individual question, largely following what Hagestad & Dannefer (2001) call *microfication*, which is the tendency to focus on immediate questions individuals might face in their daily life while overlooking broader social context that defines and frames people’s immediate experience. This microfication of aging and AI echoes a well-established western bioethical focus on individual autonomy that neglects how structural matters may predetermine older adults’ feasible choices (Ho, 2008).

For example, in the space of healthcare and direct-to-­consumer (DTC) wellness products, it is often assumed that AI technologies will give older adults who consent to use AI technologies more information regarding their health status and more individual choices around personal and health care delivery (Sharon, 2017), while sidelining the more structural aspects (such as income, education, and race) that determine individuals’ health status and health behavior. The individual focus has fueled pledges to expand AI access to older adults in the converging private, commercial, wellness, and health care spaces, for example, through affordable access for these technologies such as free health apps or mental health chatbots. Gerontechnologists tout democratization of data access by promoting a consumer logic toward health information, allowing consumers to use DTC or AI health apps to bypass gatekeepers (e.g., physicians) to control when and how they want to seek information and care according to their own priorities and wishes (Ajana, 2017). This also invites, however, an increasing risk of health misinformation in the digital space, which has been shown to be particularly high among older adults (Roozenbeek et al., 2020).

This focus of enhanced individual autonomy by AI misses structural issues that concern how much power and control other individuals and entities hold over older adults in the hyperconnected digital era (Andreotta et al., 2022). For example, there is a missing reflection on how the rapid expansion of AI technologies thwart older adult’s opportunities to make care decisions according to their goals and priorities (Mackenzie, 2014), as AI tools may gradually reduce the availability of human service and care options. The limits of an individual consent model have been written about extensively in relation to big data and AI, but infrequently in gerontology, where problems of constrained choice, practical barriers, and exclusion of other actors, such as informal caregivers and care staff, have merely been raised (Berridge & Grigorovich, 2022).

A PE perspective on AI and aging explores how decisions to invest in and deploy AI for older adults over other forms of support can exert new forms of power, inequality, and control and predetermine their available choices (Ho, 2023). It also highlights how intertwining political economic factors raise questions around to what extent older adults truly can refuse AI-powered services and retain access to human services and care. In the contexts of austerity and resource scarcity that fuel AI solutionism, the limitations of the focus on individual autonomy make urgent the need for expanded structural ethical analyses.

## AI policies

The current AI policy landscape is highly dynamic, where political, economic, legal and societal interests of different stakeholders’ groups collide. So far, two issues have been identified with respect to older adults and representation of their interests, needs and preferences in AI policy: First, general invisibility of old age in international and national policy texts from Europe and North America; and second—in those rare instances where older adults are mentioned—a stereotypical representation of their needs and potentials (Stypinska, 2023).

A recent analysis of national AI strategies in Germany, Poland, Spain, and the United Kingdom showed that older adults are generally underrepresented as a group when compared with other demographic groups, and that the diversity and inclusion of AI initiatives does not sufficiently cover age as a potential ground for discrimination by AI (Sourbati & Stypińska, 2025). This is despite the well-documented research findings on multiple forms of age-related AI bias (Chu et al., 2022; Neves et al., 2023; Stypińska et al., 2023). In international policies, the references to older adults are still relatively rare, but more nuanced. For instance, documents issued by UNESCO highlight the human rights of older persons in relation of AI (Unesco, 2022), while the policy brief of the World Health Organization warns about the risks of ageism in AI and proposes several corrective directions (WHO, 2022). Overall, however, the effective legal protection of older adults from AI discrimination and algorithmic harms is still in its infancy (Wachter, 2022). This overarching inattention to the structural rights and interests of older adults has real consequences today, and positions protection against harmful effects of AI applications as an individual issue, where older adults and their families must take action instead of enjoying protection via policy and regulation (Berridge & Ho, 2025).

To illustrate this, we draw on examples from the United States as a country that lacks both comprehensive federal data privacy protections and AI policy. In 2022, The Center for Medicare Advocacy examined use of AI by insurers in rehabilitation and home health settings and concluded that coverage decisions made using AI were more restrictive than what Medicare would have allowed (CMA, 2022). An investigation by STAT surfaced internal documents showing that a subsidiary of the largest U.S. health insurance company required employees to keep Medicare Advantage plan patients’ rehab stays within 1% of the days projected by their algorithm, regardless of Medicare coverage rules (Ross & Herman, 2023). Since then, a class action lawsuit has been filed against UnitedHealth Group, on behalf of people described as “elderly” patients wrongfully denied post-acute care, including removal from rehabilitation facilities following a stroke. The claimants argued that the company used AI to predict the level of care patients should require, resulting in generic recommendations that overrode doctor recommendations and conflict with rules on what Medicare Advantage plans must cover. At the very least, law that explicitly grants the right to an explanation for automated decision-making in this context is needed (Solaiman, 2024).

As we write, U.S. citizens’ access to benefits administered through the Social Security Administration has been thrown into limbo by Elon Musk and the Department of Government Efficiency that has massively reduced the Administration’s workforce with the promise of replacing government workers with AI tools. Efficiency is privileged over other goals at the expense of meeting material needs, and AI is presented as a way to identify waste and automate federal jobs, while the evidence shows that it wrongly cuts people off from services (Chen, 2025). In addition to the erroneous claims of fraud the current administration has spread about Social Security, the lack of responsibility to the people this impacts is striking. So too is the scale of sensitive data privacy risks with unsanctioned access attempts and promised incorporation of AI. Any lapse in governmental systems that ensure delivery of core services that older adults depend on to make rent or mortgage and purchase food and medication will be devastating. Social Security is the largest source of income for most older Americans. Gerontology’s microfication (Hagestad & Dannefer, 2001) of AI and aging has left the field on its heels in these consequential moments of a Silicon Valley takeover of government.

These cases and examples and others like it (see MacMillan & Rowland, 2024 and Lopez, 2023) illustrate how a individualistic approach to protecting the public against AI harm tends to further marginalize those older adults who might not have the resources to fight biased algorithmic decisions. Bridging the digital divide through data literacy and access expansion efforts are incommensurate and insufficient to this structural problem of power imbalance.

A PE analysis of AI opens questions such as, why are older adults nearly invisible in AI policy discourse across so many countries? What civil society infrastructure is in place to represent the interests of diverse groups of older adults in AI policy discourse? We do not lack evidence of the disconnects between older adults’ AI data privacy needs and what is offered to them (e.g., see Berridge et al., 2023; AARP, 2024); or of the widespread mistrust of AI-generated health information among older adults (Keenan et al., 2024). What we lack is a broader acknowledgement about the fact that older adults are underrepresented in AI policy making and that this has real and immediate consequences for their lives (Berridge & Ho, 2025).

## Discussion

We exemplified how the debate on AI in gerontology has predominantly focused on the individual “as the primary unit of analysis” (Estes, 2001) and discussed which complexities a PE perspective adds. Structural issues about AI are too often neglected in gerontology, overlooking how later life is systematically rendered both as a societal and individual problem that drives AI development. This concerns, for example, the ways in which AI is currently pushed as a viable solution to the challenges of population aging; a discourse on AI ethics that narrowly focuses on individual autonomy; and AI policies that currently do not take the interests, protections, and rights of older adults sufficiently into account. Each of these debates needs to be reframed from an individual and problem-centred towards a structural and empowerment-centered perspective that is in line with the roots of critical gerontology (Baars, 1991).

Such a reframing might include critical interrogations about how older adults are systematically positioned as voiceless and problematic consumers; how this positioning benefits AI development companies that develop technologies for the diverse contexts of aging; how we can rethink the restraints of an informed-choice model at the intersection of aging, care, and AI; and how older adults can be structurally protected and empowered against the current hegemony of AI development companies.

On a conceptual level, we argue toward expanding classical political economy of aging perspectives with a new materialist approach. Such an expansions is built on the acknowledgement that AI systems are neither politically neutral tools or interventions, nor passive factors that oppress and determine humans, but that humans (such as older adults, technology developers, policy makers) and nonhumans (such as data banks, algorithms, and sensor technologies) are mutually entangled in powerful assemblages and materialized practices that distribute power, capital, and inequalities in datafied and aging societies. A new materialist stance therefore expands a political economy of aging by not only asking how structural conditions of AI render old age as dependent and vulnerable but asking how boundaries between dependent and independent are drawn, and what role non-humans—such as AI technologies—play in that process.

To develop such a framework, the researchers propose a political economy of aging and AI grounded in four main concepts that can offer guidance for further critical and material exploration of the relationships between aging and AI:

First, a focus on the *techno-bio-politics of aging* that starts from the assumption that technology is almost universally interconnected with any political problem or societal domain that concerns aging and asks how aging is governed under rapidly changing technological conditions (see Lipp & Maasen, 2022). It has already been shown that a techno-biopolitical regime of AI tends to make older adults responsible for managing and investing in their health and long-term care through data work and data management (Lipp & Peine, 2024). Such quantified responsibilization becomes even more powerful the more older adults’ engagement with AI is positioned as a prerequisite for securing their contribution to economic growth, which many believe to be AI’s most relevant potential. A materialist inspired PE approach might ask new questions about how aging is governed with and through AI, and how humans and non-humans contribute together to making (and challenging, changing, or refusing) these complex systems of power that currently drive AI development and implementation in the context of aging.

Second, we propose to focus on the logics of *data capital and data extraction* that are inherent in the development and implementation of AI in the context of aging. Research has already highlighted that due to the lack of availability of data on older adults for AI development (Chu et al., 2022), unique age-data has emerged as a profitable market for companies (Gallistl et al., 2024). A PE perspective makes visible these relationships of data extraction and profit generation that underpin the datafication of long-term care and tends to understand the absence of data on older adults as a possibility of profit creation rather than a chance of fostering older adults’ inclusion (Gallistl et al., 2024). A materialist inspired PE approach might also make visible the situated and material conditions of the current AI hype—the data banks, the energy demands, the invisible human labor that goes into AI systems—to get a better grasp how AI is not just about abstract promises, but a material reality that often builds on the exploitation of users, workers, and natural resources.

Third, the researchers propose to use the *microfication* of ethical issues in relation to AI for critical-gerontological analysis. Instead of the current focus on individual autonomy and agency in relation to AI, the researchers propose a relational lens of autonomy (Ho, 2023) that highlights how intertwining political economic factors raise questions around to what extent older adults truly have power and authority over decisions significant to the direction of their lives and care pathways. This requires attention to constrained choice and the power asymmetries in care relationships, including those between older adults and healthcare institutions (Ho, 2023). It also requires critical gerontology to ask new questions around the nature and limits of human agency in a digitized world. A critical and material approach might highlight how agency is not seen as a characteristic of human action, but emerges in the complex interplay between human and nonhuman actors (Wanka & Gallistl, 2018). For a critical exploration of the AI-aging-enterprise, this might mean to consider how older adults’ agency might be strengthened against the dominant power of AI systems and the companies that develop them, but also how agency (and empowerment) can be achieved *together-with* technological infrastructures and AI systems.

Fourth, more attentiveness to the policy needs and interests of older adults in the political economy of AI is needed. Current AI policies do little to acknowledge the need for protection of older adults against the use of AI systems in sensitive settings, such as health or long-term care (Berridge & Ho, 2025), or the ageism that is inherent in much of AI development. For example, Stypinska (2023) ably shows how age biases are incorporated into algorithms and digital data sets, into discourses and cultural imaginaries around AI, or implementation practices in real-world settings. These and other forms of AI ageism (Chu et al., 2022; Neves, 2023; Stypinska, 2023) need to be addressed first because they undermine all remaining efforts to bring attention to the interests, needs and preferences of older adults in relation to AI. The lack of policy protections against AI ageism will be particularly harmful for those older adults who lack financial resources, digital competencies, social support, or experience multiple forms of marginalization.

Lastly, we want to acknowledge that the materialist-enhanced PE approach we put forward in this article remains conceptually open, and subject to debate and interpretation. Reconciling structuralist and new materialist perspectives, and asking about the critical potential of material gerontology, has already been the subject of lively debate in gerontology (Höppner, 2023) and beyond. With this article, we invite others to think further about the critical potential of a still-developing material gerontology—one that highlights the political-economic entanglements shaping aging in a digitized and datafied world and imagines ways of changing them.

## Data Availability

The authors do not report data and therefore the pre-registration and data availability requirements are not applicable.

## Footnotes

1 Marx himself was probably one of the first to suggest detailed analysis of the dynamics of individual technologies as part of social and historical analysis (Rosenberg, 1982). But in the political economy of aging, like in many other fields of the social sciences, his historical materialism at large has been more influential than his work on technology.

2 Another instance of technological determinism entering the political economy of aging, though never explicitly, is Riley’s notion of structural lag, in which she argues that societal conditions for growing old lag behind changes in the actual practices of aging (Riley and Riley, 1989). In developing this concept, she was strongly influenced by Ogburn’s notion of cultural lag, which explains cultural change in terms of adaptations or maladaptations to technological change. Ogburn’s (1950) idea is often regarded as a foundational text of technological determinism (Rammert, 2016).
